# The Quarantine Appropriations

**Published:** 1899-06

**Authors:** 


THE QUARANTINE APPROPRIATIONS.—The Twenty-sixth Legislature was exceedingly liberal with the Quarantine Department, having made an appropriation for it for the next year of $80,500, and for the second year $45,500. I may state that this is about double the amount they allowed the lamented Swearingen, and the streak of generosity on the part of the "Solons" is rather hard to account for. It is to be observed that no additional duties were made incumbent on the State Health Officer. Of the amounts appropriated, however, $15,000 is for the purchase of a floating disinfecting plant at Galveston, to take the place of the disinfecting warehouse built a few years ago at considerable cost on Pelican Spit, and which is now high and dry. Ten thousand dollars was intended also for the purchase of sites at Texarkana, Waskom, Logansport,

Sabine River Crossing and other points, and to build detention accommodations for use in times of yellow fever scare, and $8000 annually, for supplies for detained persons, and to pay inspectors and guards. The last two items, aggregating $18,000, the Governor promptly vetoed. Some time when a lot of women and children are held as "suspects" at some camp where no shelter other than a leaky tent is provided by the great State of Texas, as was the case last year, and when the "cold chilling winds of November" come, some woman or child is going to die from the effects of exposure and the State Health Officer will have a law suit on his hands. Our people are great on the "save"; they save at the spigot and lose at the bung.

